# Expanding molecular diagnostic coverage for tuberculosis by combining computer-aided chest radiography and sputum specimen pooling: a modeling study from four high-burden countries

**DOI:** 10.1186/s44263-024-00081-2

**Published:** 2024-08-01

**Authors:** Andrew James Codlin, Luan Nguyen Quang Vo, Tushar Garg, Sayera Banu, Shahriar Ahmed, Stephen John, Suraj Abdulkarim, Monde Muyoyeta, Nsala Sanjase, Tom Wingfield, Vibol Iem, Bertie Squire, Jacob Creswell

**Affiliations:** 1 Friends for International TB Relief, Hanoi, Viet Nam; 2 Karolinska Institutet, Stockholm, Sweden; 3 Stop TB Partnership, Geneva, Switzerland; 4 icddr,b, Dhaka, Bangladesh; 5 Janna Health Foundation, Yola, Nigeria; 6 Centre for Infectious Disease Research in Zambia, Lusaka, Zambia; 7 Liverpool School of Tropical Medicine, Liverpool, United Kingdom; 8 Liverpool University Hospitals NHS Foundation Trust, Liverpool, United Kingdom

**Keywords:** GeneXpert, Pooling, AI, CAD, X-ray, CXR, ACF, Active case finding, Tuberculosis diagnosis

## Abstract

**Background:**

In 2022, fewer than half of persons with tuberculosis (TB) had access to molecular diagnostic tests for TB due to their high costs. Studies have found that the use of artificial intelligence (AI) software for chest X-ray (CXR) interpretation and sputum specimen pooling can each reduce the cost of testing. We modeled the combination of both strategies to estimate potential savings in consumables that could be used to expand access to molecular diagnostics.

**Methods:**

We obtained Xpert testing and positivity data segmented into deciles by AI probability scores for TB from the community- and healthcare facility-based active case finding conducted in Bangladesh, Nigeria, Viet Nam, and Zambia. AI scores in the model were based on CAD4TB version 7 (Zambia) and qXR (all other countries). We modeled four ordinal screening and testing approaches involving AI-aided CXR interpretation to indicate individual and pooled testing. Setting a false negative rate of 5%, for each approach we calculated additional and cumulative savings over the baseline of universal Xpert testing, as well as the theoretical expansion in diagnostic coverage.

**Results:**

In each country, the optimal screening and testing approach was to use AI to rule out testing in deciles with low AI scores and to guide pooled vs individual testing in persons with moderate and high AI scores, respectively. This approach yielded cumulative savings in Xpert tests over baseline ranging from 50.8% in Zambia to 57.5% in Nigeria and 61.5% in Bangladesh and Viet Nam. Using these savings, diagnostic coverage theoretically could be expanded by 34% to 160% across the different approaches and countries.

**Conclusions:**

Using AI software data generated during CXR interpretation to inform a differentiated pooled testing strategy may optimize TB diagnostic test use, and could extend molecular tests to more people who need them. The optimal AI thresholds and pooled testing strategy varied across countries, which suggests that bespoke screening and testing approaches may be needed for differing populations and settings.

**Supplementary Information:**

The online version contains supplementary material available at 10.1186/s44263-024-00081-2.

## Background

When the World Health Organization (WHO) recommended the Xpert MTB/RIF assay (Cepheid; Sunnyvale, CA, USA) for diagnosis of tuberculosis (TB) in 2010, it was heralded as a game-changer [1]. This novel technology of cartridge-based nucleic acid amplification test (NAAT) ushered in a new era of progress in TB diagnostics and was followed shortly thereafter by the second molecular WHO-recommended rapid diagnostic test (mWRD), the Molbio Truenat [2]. Today, the TB diagnostic pipeline is healthier than ever, with at least 35 other NAATs for diagnosing TB in development [3].

Despite the bright future, only 47% of people newly diagnosed with TB received a mWRD as their initial test in 2022 [4, 5]. Meanwhile, most persons with TB were still diagnosed by smear microscopy, the same method Robert Koch used to isolate *M. tuberculosis* as the etiologic agent in 1882 [6]. Among the many causes for this diagnostic coverage gap, a major reason is cost [7, 8]. Despite recent price reductions of consumables and reagents [9], ensuring universal mWRD coverage globally may cost over $1 billion per year [10, 11].

To mitigate high mWRD costs, a screening step can be used to rule out people with a low probability of TB disease [12]. Among various options, chest X-ray (CXR) has become the screening tool of choice in many settings due to the ability to identify the large cohort of asymptomatic people with TB [13, 14]. More recently, computer-aided detection (CAD) and artificial intelligence (AI) platforms to support CXR reading, such as qXR (Qure.ai; Mumbai, India), CAD4TB (Delft Imaging; Delft, the Netherlands), and other platforms, have gained popularity with value propositions ranging from capacity creation to overcome the lack of trained human readers to workload reduction through triaging of normal CXR images [15]. For individuals 15 years and above, CAD/AI may be used in place of human reading for screening and triage, and working alongside human readers to help automate and standardize interpretation [12]. Several evaluations of CAD/AI compared to human readers have shown that the technology performs as well or better than expert human readers [16–18]. CAD/AI offers a key advantage over human readers through its provision of a continuous abnormality score (ranging from 0–1 or 0–100) which confers a likelihood of TB among people screened, unlike humans who will produce a dichotomous outcome (TB suggestive or not), and grants greater flexibility to tailor follow-on testing to limited public health budgets [19].

Another recent process innovation to address the high costs of laboratory tests in resource-constrained settings that was effectively employed during the COVID-19 pandemic is specimen pooling. This method involves mixing specimens for a two-step hierarchical diagnostic algorithm that foregoes individual testing in the event of a negative pooled sample [20]. While initial TB pooling studies had found that dilution of the bacterial load can lead to lower levels of detection and potentially missed diagnoses [21], more recent evaluations have reported sensitivities of 98–100% with the Xpert MTB/RIF Ultra assay (Xpert Ultra) [22–24]. This has led to reported reductions in TB testing costs of 57–87% depending on the pool size [25]. A recent cost-effectiveness analysis reported a 34.9% decrease in costs when comparing pooled to individual Xpert testing [26].

The benefits of pooling are proportional to the prevalence of the disease in the target population. The lower the prevalence, the higher the theoretical savings. This raises the utility of pooling in high-throughput, low-yield approaches, such as active case finding (ACF) campaigns where large numbers of individuals need to be screened and tested to detect a person with TB. ACF is an important component of all high-burden countries’ national TB response and is critical to reach those people who are less likely or able to get care in public facilities. However, it is more expensive to conduct outreach, and ways to reduce costs are urgently needed to reach all persons with TB, especially those with subclinical TB [27, 28].

Here, we evaluated the theoretical impact of using AI outputs to inform different pooling strategies based on Xpert testing data collected during ACF campaigns in four high TB burden settings with the goal of modeling diagnostic savings and theoretical expansion of access to mWRDs.

## Methods

### Study design

This was a retrospective analysis of ACF campaigns using AI probability scores to model the incremental reduction in Xpert cartridge consumption.

### Data sources

Data were obtained from four implementers located in high TB burden countries of Bangladesh, Nigeria, Viet Nam, and Zambia. These data consisted of aggregate AI abnormality scores and Xpert test results from community- and facility-based case-finding campaigns conducted between 2014 and 2017 in Bangladesh and 2022 and 2023 in all other countries. These campaigns targeted a heterogeneous mix of vulnerable populations particular to each ACF setting and country.

All countries used CXR with slightly different modalities to screen for TB but presumptive TB was identified by either symptom screening or an abnormal chest radiograph. Screening methods for each country are summarized here. In Bangladesh, standalone screening centers supported referrals of health-seeking symptomatic individuals in outpatient care departments of public and private sectors to conduct facility-based CXR screening and Xpert testing in Dhaka [29]. In Nigeria, mobile teams conducted active outreach events in remote rural areas using ultra-portable X-rays among people with limited access to healthcare services, such as pastoralists and nomadic populations using verbal symptom screening and CXR in parallel [30]. Viet Nam delivered community-based ACF campaigns in rural and urban areas focused on household contacts, older persons, urban poor, and people with a history of TB with verbal symptom screening and CXR in parallel [31, 32]. Zambia used a portable X-ray system in health facilities to screen clinic attendees and household contacts who were symptomatic [33]. Nigeria and Viet Nam used qXR v3 (Qure.ai, India), and Zambia used CAD4TB7 (Delft Imaging, The Netherlands) for the generation of the AI probability score. Bangladesh used qXR v3 and CAD4TB6 for the same dataset, but only qXR v3 results from the former were used due to concordance with the software used in Nigeria and Viet Nam. Xpert MTB/RIF (Bangladesh and Nigeria) or the newer Xpert MTB/RIF Ultra assay (Viet Nam and Zambia) was used for diagnostic testing of distinct individuals in all countries. Both CAD4TB and qXR interpret CXR images as DICOM files and produce a TB abnormality score that confers a likelihood that the individual has TB and therefore should be tested. CAD4TB provides a score ranging from 0 to 100, while qXR produces a score ranging from 0 to 1 [34].

### Model structure

Each participating country provided Xpert testing data aggregated by deciles (*D*_*m*_, where *m* = 1 to 10) of AI probability scores in the range of 0–100 for CAD4TB, i.e., *D*_*1*_: AI score = 0–9, *D*_*2*_: 10–19 …, and, similarly, 0–0.99 for qXR. We calculated the positivity rate (*p*_*m*_) for each *D*_*m*_. We assigned testing thresholds for each country based on the decile (*D*_*m*_) where ~95% of cases would be detected (*C*) with Xpert testing starting at that decile to have only ~ 5% missed cases (*M*) as a result of not employing Xpert testing in the previous deciles (Additional file 1: Table S1, S2, S3). The 5% rate of missed TB cases was chosen to acknowledge the real-life resource limitations of testing all people with Xpert, while still maintaining a high level of detection.

**Fig. 1:**
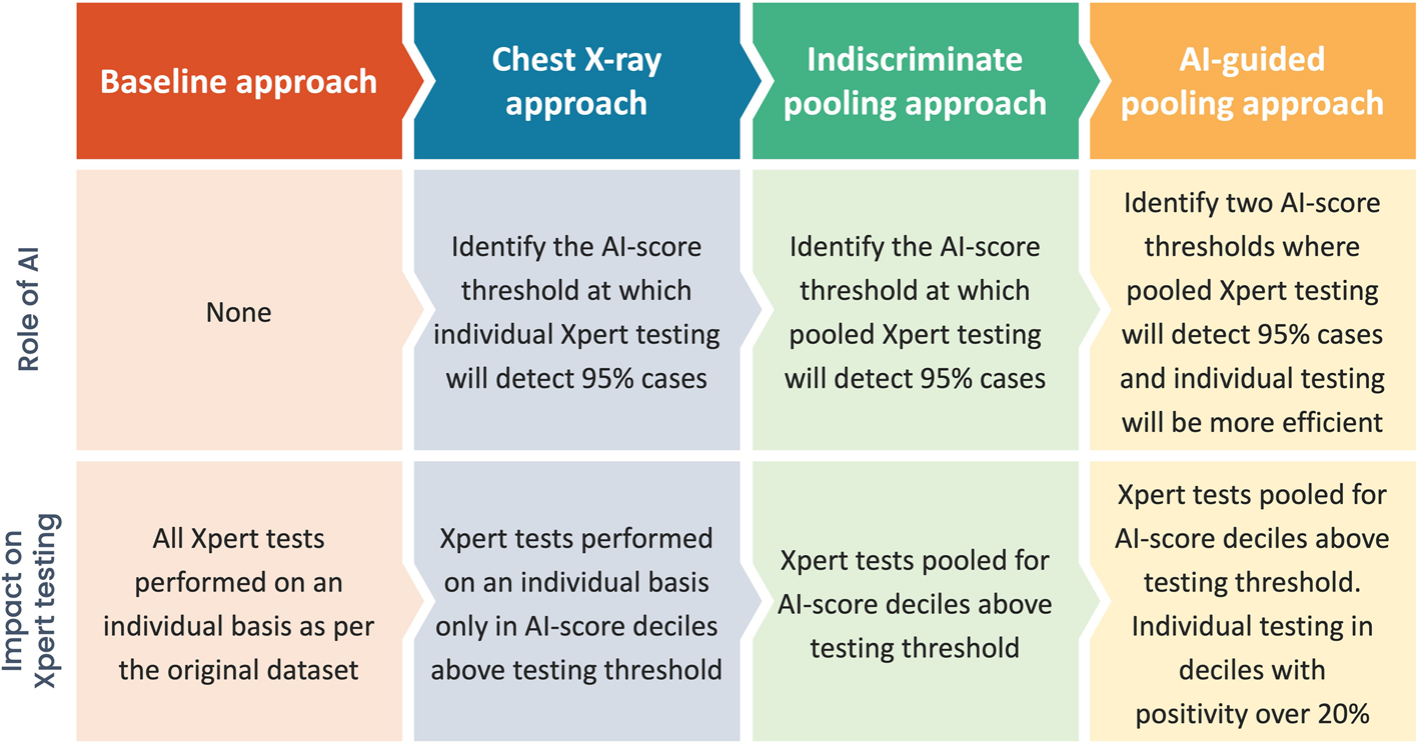
The framework for analysis of the role of AI and its impact on Xpert testing

To model the theoretical savings, we calculated the theoretical number of diagnostic tests per person needed to achieve the same positivity through pooling for a two-step hierarchical testing strategy [35]. The difference between the total actual number of individual tests performed and the theoretical number of tests per person when employing the pooled testing strategy represented the number of tests saved. For the primary analysis, we assumed a pool size of four based on prior studies and that both individual and pooled testing were 100% sensitive and specific [36, 37]. We then modeled four ordinal screening and testing approaches with increasing complexity to estimate incremental savings compared to the previous approach (Fig. 1):

1. Baseline approach: all people with presumptive TB receive individual Xpert tests as per the original datasets;
2. CXR approach: individual Xpert tests in all deciles for which *ΣM* ~ 5%;
3. Indiscriminate pooling approach: pooled Xpert tests in all deciles for which *ΣM* ~ 5%;
4. AI-guided pooling approach: a combination of pooled and individual Xpert tests in all deciles for which *ΣM* ~ 5%, with individual Xpert testing in deciles with a *p*_*m*_ ≥ 20%. The 20% cutoff was chosen based on the peak level of savings that pooling would generate at various levels of positivity based on the outputs of our model.

While we aimed to achieve a *ΣC* ~ 95% (≡ *ΣM* ~ 5%) for screening and testing approaches, the actual values were 95.7% (Bangladesh), 95.3% (Nigeria), 94.9% (Viet Nam), and 96.7% (Zambia).

### Data analysis

We described the number of tests performed, positive results, and positivity rates in total and for each AI-score decile. We further calculated the theoretical number and proportion of tests saved incrementally between each screening and testing approach and cumulatively over the baseline. To characterize the optimal approach in each country, we identified the deciles below which testing could be foregone and the deciles at which individual testing instead of pooled testing would save tests. The number of tests saved was multiplied by the current price for an Xpert Ultra cartridge ($7.97) to calculate crude cost savings and subsequently divided by the number of positive test results for a unit-cost estimate per person diagnosed with TB [9]. To offer an alternative perspective to cost savings, we estimated the ratio of additional tests that could be performed with the savings over the theoretical number of tests needed for the current cohort as a measure of the extent to which access to mWRDs could have been expanded by employing the optimal screening and testing approach.

For the sensitivity analyses, we modeled using pool size of three [22] and relaxed the 100% sensitivity and specificity assumption of the pooling method to 95% and 98%, respectively, based on systematic review findings from pooling with Xpert Ultra as Xpert MTB/RIF will be discontinued in 2024 using the datasets from all countries [23]. We used binGroup2 package in R for the analysis and have publicly availed all data and analysis code [38, 39].

## Results

### Dataset characteristics

**Fig. 2:**
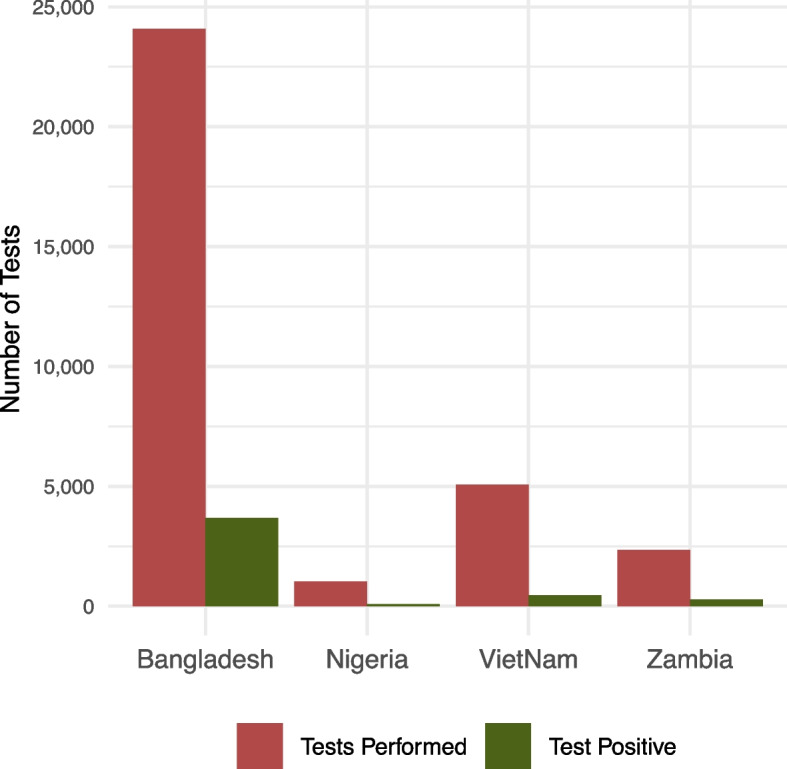
Xpert testing data for the four countries. *Legend.* The percentages are reported with reference to number of tests performed. The Xpert positivity ranged from 8% in Viet Nam to 15% in Bangladesh.

In the two facility-based ACF settings, Bangladesh and Zambia performed 24,079 and 2353 Xpert tests with a matching corresponding AI result, respectively. In the community-based ACF settings, Nigeria performed 1021 and Viet Nam performed 5074 tests. Positivity rates were 15.3% (3679/24,079) in Bangladesh, 11.6% (273/2353) in Zambia, 9.0% (455/5,074) in Viet Nam, and 8.3% (85/1021) in Nigeria (Fig. 2).

**Fig. 3:**
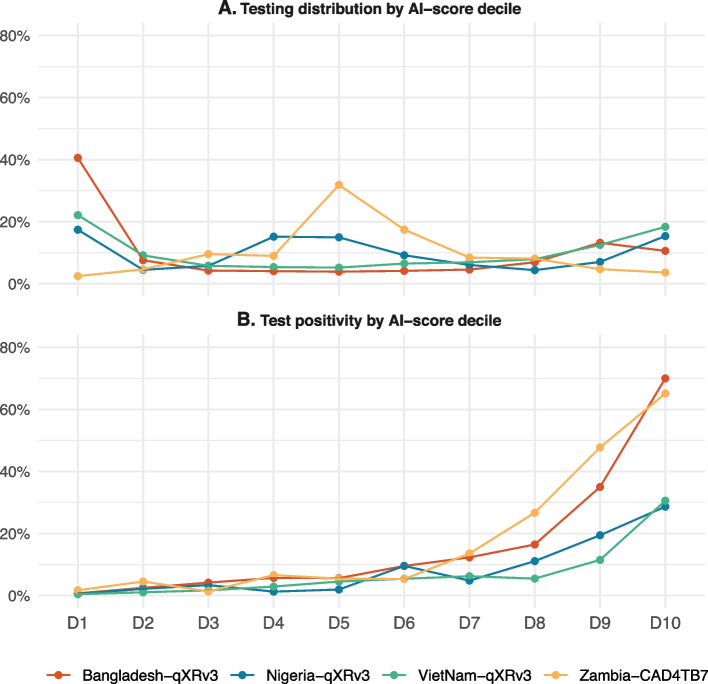
Distribution of tests performed and test positivity across the AI-score deciles for the four countries. **A** Testing distribution by AI-score decile refers to the percentage of total tests performed within each AI-score decile. **B** Test positivity by AI-score decile shows the proportion of positive results in each AI-score decile. The decile labels (D1 to D10) represent AI-score deciles; D1–D10 for 0–0.99 in 0.1 intervals for qXR score and D1–D10 for 0–99 in 10-point intervals for CAD4TB score

In terms of testing distribution by AI-score decile, most countries exhibited similar U-shaped patterns except Zambia (Fig. 3A). In Zambia, almost half (49.3%) of the tests were conducted in *D*_*5*_*–D*_*6*_. Meanwhile, 40.6% of Xpert tests in Bangladesh had an AI score in the lowest decile (*D*_*1*_), which was higher than Viet Nam (22.1%), Nigeria (17.4%), and Zambia (2.5%). Meanwhile, the testing rate in the AI-scores *D*_*4*_*–D*_*5*_, were higher in Nigeria (*D*_*4*_:15.3% and *D*_*5*_:15.0%) compared to Bangladesh (*D*_*4*_:4.1% and *D*_*5*_:3.9%) and Viet Nam (*D*_*4*_:5.4% and *D*_*5*_:5.2%).

In terms of positivity by AI-score decile (Fig. 3B), the two facility-based ACF sites exhibited higher rates than the community-based counterparts. In Bangladesh, positivity was higher in the high AI-score deciles of *D*_*7*_*–D*_*10*_ than in the other countries, with rates increasing from 12.3 to 70.0%. Zambia exhibited a similar pattern with a lower peak with rates, ranging from 13.6 to 65.1% for *D*_*7*_ and *D*_*10*_, respectively. In comparison, the respective positivity of *D*_*7*_ and *D*_*10*_ only rose from 4.8 to 28.7% in Nigeria and from 6.3 to 30.6% in Viet Nam.

### Tests cost savings in the model output

All incremental screening and testing approaches resulted in additional savings except the indiscriminate pooling approach in Bangladesh and are presented for each country in Table 1. In Bangladesh, the CXR approach resulted in savings of 52.5% over baseline. Based on model parameters, these savings were realized at an AI threshold of 0.30–0.39, i.e., foregoing testing for *D*_*1*_*–D*_*3*_ and individually testing everyone in higher deciles. Indiscriminately pooling all persons in *D*_*4*_*–D*_*10*_ actually resulted in excess of 2.0% of tests over the CXR approach. Using AI to indicate individual testing for high AI-score deciles *D*_*9*_*–D*_*10*_ reversed this trend and increased cumulative savings to 61.5%.

**Table 1:** Incremental and cumulative savings by country

	Tests used	Incremental savings		Cumulative savings	
	*N*	*N*	%	*N*	%
Bangladesh					
Baseline approach	24,079				
CXR approach	11,448	12,631	52.5%	12,631	52.5%
Indiscriminate pooling approach	11,676	− 228	− 2.0%	12,403	51.5%
AI-guided pooling approach	9262	2414	20.7%	14,817	61.5%
Zambia					
Baseline approach	2353				
CXR approach	1960	393	16.7%	393	16.7%
Indiscriminate pooling approach	1352	608	31.0%	1001	42.5%
AI-guided pooling approach	1158	194	14.3%	1195	50.8%
Nigeria					
Baseline approach	1021				
CXR approach	738	283	27.7%	283	27.7%
Indiscriminate pooling approach	459	279	37.8%	562	55.0%
AI-guided pooling approach	434	25	5.4%	587	57.5%
Viet Nam					
Baseline approach	5074				
CXR approach	2917	2,157	42.5%	2157	42.5%
Indiscriminate pooling approach	2110	807	27.7%	2964	58.4%
AI-guided pooling approach	1955	155	7.3%	3119	61.5%

*CXR* Chest X-ray, *AI* Artificial intelligence. Incremental savings are the test saved compared to the previous approach. Cumulative savings are the tests saved compared to the baseline approach

The model assumes pool sizes of 4 with both pooling and individual testing sensitivity and specificity of 100% for a testing threshold resulting in missed cases of 4.4%, 4.7%, and 5.1% for Bangladesh, Nigeria and Viet Nam using qXR v3, respectively, and 3.3% for Zambia using CAD4TB v7. Incremental savings indicate the difference from the prior testing approach in the table (e.g., indiscriminate pooling approach compared to CXR approach or AI-guided pooling approach compared to indiscriminate pooling approach), whereas cumulative savings are calculated against the baseline approach.

In Zambia, the CXR approach yielded savings of 16.7% over baseline at an AI threshold of 0.3. Indiscriminately pooling all persons in *D*_*4*_ and higher produced incremental savings of 31.0% and cumulative savings over the baseline of 42.5%. Per AI guidance, testing reverted to an individual basis in *D*_*8*_*–D*_*10*_ to yield additional savings of 14.3% for cumulative savings of 50.8%.

The CXR approach in Nigeria showed an initial savings of 27.7% in testing at an AI threshold of 0.3 above which indiscriminate pooling reduced an incremental 37.8% in testing for a cumulative savings of 55.0% over baseline. There were diminishing returns from the AI-guided pooling approach as switching to individual testing in *D*_*10*_ led to incremental and cumulative savings over the baseline were only 5.4% and 57.5%, respectively.

**Fig. 4:**
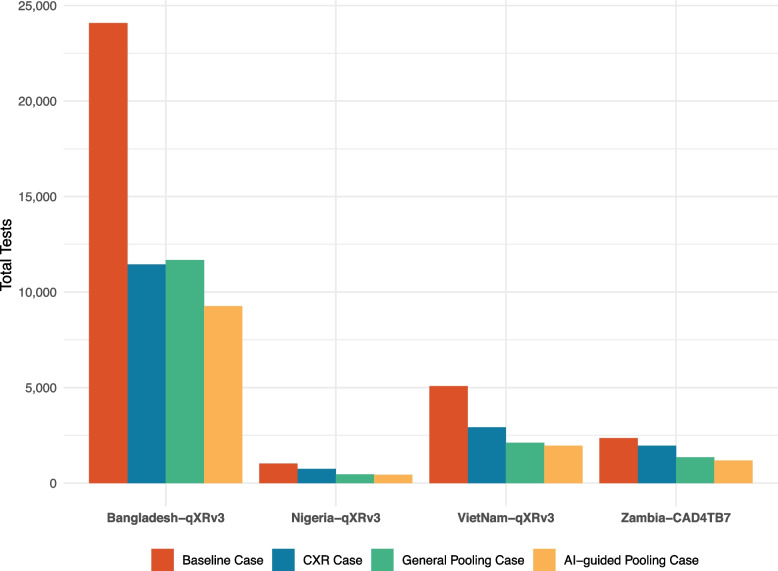
Tests performed under different approaches in the four countries. *Legend.* The model assumes pool sizes of 4 with both pooling and individual testing sensitivity and specificity of 100% for a testing threshold resulting in missed cases of 4.4%, 4.7%, and 5.1% for Bangladesh, Nigeria, and Viet Nam using qXR v3, respectively, and 3.3% for Zambia using CAD4TB v7. The percentages are reported with reference to the baseline approach for the respective countries

In Viet Nam, the CXR approach reduced testing by 42.5% at an AI threshold of 0.4. Meanwhile, indiscriminate pooling above the AI threshold saved an incremental 27.7% in testing over the CXR approach for a cumulative savings of 58.4% over baseline. Similar to Nigeria, the AI-guided pooling approach indicated individual testing in *D*_*10*_ which added 7.3% in incremental test reductions for a cumulative savings of 61.5% (Fig. 4).

In Bangladesh, the number of tests needed declined from 24,079 to 9262 for savings of 12,403–14,817 tests. This translated to $98,852–$118,091 in crude costs or $26.87–$32.10 per person with TB diagnosed (Table 2). Alternatively, these savings could be redeployed for an expansion of mWRD access of 110–160% with existing public health resources. In Zambia, the number of tests dropped from 2353 to 1158 for savings of 393–1195 tests. This implies cost savings of $3132–$9524 or $11.47–$34.89 per person with TB for a theoretical expansion of mWRD access of 20–103%. Based on test volume reductions from 1021 to 434 in Nigeria, the total test and cost savings ranged from 283 to 587 and $2256–$4678 or $26.54–$55.04 per person with TB. This represented 38–135% in potentially greater access to mWRD. Lastly, in Viet Nam the number of tests needed fell from 5074 to 1955 for 2157–3119 tests saved corresponding to crude savings of $17,191–$24,858 or $37.78–$54.63 per person with TB. This represented a potential mWRD expansion of 74–160%.

**Table 2:** Crude total and per-TB detection cost savings and mWRD access expansion by country

	Crude cost savings ($)		Per-TB cost savings ($)		mWRD access expansion	
	Incremental	Cumulative	Incremental	Cumulative	Incremental	Cumulative
Bangladesh						
Baseline approach						
CXR approach	100,669	100,669	27.36	27.36	110.3%	110.3%
Indiscriminate pooling approach	− 1817	98,852	− 0.49	26.87	− 2.0%	106.2%
AI-guided pooling approach	19,240	118,091	5.23	32.10	26.1%	160.0%
Zambia						
Baseline approach						
CXR approach	3132	3132	11.47	11.47	20.1%	20.1%
Indiscriminate pooling approach	4846	7978	17.75	29.22	45.0%	74.0%
AI-guided pooling approach	1546	9524	5.66	34.89	16.8%	103.2%
Nigeria						
Baseline approach						
CXR approach	2256	2256	26.53	26.53	38.3%	38.3%
Indiscriminate pooling approach	2224	4479	26.16	52.70	60.8%	122.4%
AI-guided pooling approach	199	4678	2.34	55.04	5.8%	135.3%
Viet Nam						
Baseline approach						
CXR approach	17,191	17,191	37.78	37.78	73.9%	73.9%
Indiscriminate pooling approach	6432	23,623	14.14	51.92	38.2%	140.5%
AI-guided pooling approach	1235	24,858	2.72	54.63	7.9%	159.5%

*CXR* Chest X-ray, *AI* Artificial intelligence, *Mwrd* Molecular WHO-Recommended Rapid Diagnostic

Incremental savings indicate the difference from the prior case, whereas cumulative savings are calculated against the baseline approach. Crude cost savings are based on a cost of $7.97 per Xpert Ultra cartridge. Per-TB cost savings are based on a number of positive test results of 3679 in Bangladesh, 273 in Zambia, 85 in Nigeria, and 455 in Viet Nam. mWRD access expansion was calculated by dividing the number of cartridges saved by the number of cartridges used in each individual screening and testing approach.

The sensitivity analyses did not show a substantial change in the results. Across all different scenarios, the change in savings against the primary case (pool size of three with pooled sensitivity and specificity of 1) ranged from − 9.4% to + 7.6%. Testing in smaller pools increased the number of tests used. Reducing pooling sensitivity reduced test usage and so did increasing pooling specificity, which will result in an increase in missed cases and false positives, respectively, for the overall two-step hierarchical testing algorithm. (Additional file 1: Table S4) In Bangladesh, we compared savings between qXRv3 and CAD4TBv6 and found that differences were small (0.3%). CAD4TB scores resulted in 9200 tests in the AI-guided pooling approach compared to 9262 in qXRv3 against the baseline case of 24,079 tests. (Additional file 1: Table S5).

## Discussion

Our modeling study results show that the combined use of computer-aided chest radiography and pooling may achieve compounding effects to achieve significant savings in diagnostic consumables that could be redeployed to increase the global coverage of mWRDs. We further found that the substantial heterogeneity in AI thresholds and the impact of AI scores on subsequent pooling across the different settings will require differentiated deployment of these two innovations in order to optimize the potential gains. However, while countries and settings may differ, it appears that the various screening and testing approaches particularly the AI-guided pooling approach may be able to achieve consistent results between the most advanced software platforms.

Across the different countries, settings, and approaches, the combination of using CXR with AI scores to inform decisions on pooling sputum produced cumulative savings on testing of 50.8–61.5% over baseline compared to universal testing of all individuals with signs of TB. In 2022, Bangladesh tested only 20% of people with TB with mWRDs as the first-line diagnostic test. While the gap was smaller in Nigeria, Zambia, and Vietnam, three in ten persons with TB were not diagnosed using molecular diagnostics [4]. Our results suggest that more than twice as many people could be tested for the same diagnostic test costs using CXR and pooling as compared to testing all presumptive individuals.

A major contributor to this potential capacity expansion was the use of AI and CXR. As the data of all countries originated from ACF campaigns rather than prevalence surveys, each dataset included an inherent level of preselection or pre-screening to raise the TB prevalence in the sample. This pre-filtering may have consisted of targeting highly vulnerable populations, such as contacts (Zambia), deploying in high-yield settings such as health facilities (Bangladesh), or having a previous binary read of the CXR by a potentially inexperienced human reader (Viet Nam) [17]. Despite these methods of preselection, our study once again highlighted the well-documented effectiveness of CXR for screening and triaging for TB [40–43]. Beyond that, we observed incremental savings by leveraging the AI’s quantitative output to optimize CXR interpretation and forego testing in lower AI-score deciles. This approach was particularly useful in settings with a high testing proportion in the lowest decile such as Bangladesh and Viet Nam, the first screening and testing approach generated already generated cumulative savings of 52.5% and 42.5%, respectively. This optimization of testing volumes through computer-aided chest radiography was also concordant with the available evidence [18, 30].

Our model suggested that in each country, pooling could be effective for generating additional savings in testing. However, our model also evinced differences across both pooling strategies based on the populations screened and the results of the CXR and AI. For instance, in the facility-based settings of Bangladesh and Zambia, 65–70% of individuals in the highest decile had bacteriologically confirmed TB compared to only 29–31% in Nigeria and Viet Nam’s community-based ACF cohort. This dichotomy was reflected by the pooling approach, as the AI-guided pooling, i.e., reversion to individual testing in high deciles, continued to generate substantial savings in the facilities, while the model exhibited substantial diminishing returns in the low-yield community setting. Interestingly, while indiscriminate pooling saved tests in three countries, in Bangladesh it actually increased testing unless an AI-guided pooling approach was used. In Bangladesh, where reads for both qXR and CAD4TB were available, the performance on both platforms was highly concordant. However, each required adjustment of the testing threshold, highlighting the need for end-user optimization based on the local epidemiology and platforms used. The approaches may be further optimized based on where sample volumes allow more pools. In one such additional approach, the AI-guided cohort pooling case resulted in up to 2.5% additional savings over the AI-guided pooling case. (Additional file 1: Table S5) Nevertheless, these findings inspire confidence in the high precision that characterizes many of today’s commercial AI solutions for TB [16].

This variability was encountered in different areas of our study and represented its key strength. For example, in contrast to the high savings achieved through the CXR approach in Bangladesh and Viet Nam, savings in Nigeria and Zambia were only 28% and 17%, respectively. Yet, both countries differed substantially in the additional gains from each incremental pooling approach. The testing distribution patterns also exhibited discordance, as all countries exhibited a U-shaped curve except Zambia, or in positivity by decile, whereby reversion to individual testing of the AI-guided pooling approach occurred in *D*_*8*_ for the Zambian dataset, in *D*_*9*_ for the data from Bangladesh and *D*_*10*_ in Nigeria and Viet Nam. Given the variation among the data we analyzed, any program implementing such an approach should consider individual results before deciding on a specific AI and pooling strategy. However, the results suggest that efficiency gains are likely across different settings. Particularly with respect to the optimal application of AI and its continuous outputs, there has been much discussion about using different AI threshold scores. However, the growing evidence base suggests variability in AI performance across demographic, clinical, or behavioral characteristics of the population, screening setting, or even radiography equipment, which underscores the well-documented need for local calibration and threshold setting [18, 19, 44]. Similarly, while general pooling can save costs, optimizing a pooling strategy will depend on the use of local data, capacities, and established practices.

Our study has a limitation in that modeled results are fraught with assumptions about performance and each situation will differ. AI technology changes rapidly and our data comes from different time periods with different screening approaches, but the results are generally similar in improving efficiency with AI. In the primary analysis, we assume perfect sensitivity and specificity for both pooling and individual testing, which is unlike real-world performance. While we attempt to mitigate this limitation in the sensitivity analysis by varying the pooling parameters, we do not model the overall performance of the diagnostic algorithm, e.g., the overall sensitivity and specificity of a combination of no testing, individual testing, and pooled testing in the AI-guided pooling case. This will also impact the overall number of missed cases in the algorithm, leading to continued transmission and potential mortality. False positive results would increase testing costs and workload. We do not expect a substantial impact on the number of tests given the superior performance of Xpert Ultra in both individual and pooled testing documented previously and encourage future prospective evaluations to factor in these considerations, in addition to other implementation challenges. None of these studies used culture to quantify how many people would have been missed due to Xpert Ultras’ imperfect sensitivity, but our analysis was focused on optimizing molecular testing. Importantly, our model assumes AI is available to interpret CXR images but does not have any other cost for the technology which has a cost, as do human readers. Costing studies on AI and CXR are urgently needed as programs consider adopting the technology and so are cost-effectiveness analyses that capture the complete costs of pooling interventions. Future work should also consider different scenarios (e.g., paucibacillary samples, children) which may have different results from pooled testing approaches. Lastly, although the data are based on real-world ACF activities they did not contain the daily distribution of people with presumptive TB and their abnormality scores. Hence, for this analysis, we assumed 100% completeness in pool sizes with four sputum specimens per pool. Pooling decisions based on abnormality scores such as the AI-guided pooling approach would require that these data be included in the laboratory order form, and on occasion require pools of two or three specimens, which might impact overall savings and complicated pooling rules may cause confusion in laboratories. However, we conducted this sensitivity analysis, which also showed that the impact on overall savings was small.

The real-world readiness for computer-aided chest radiography-informed pooling in real-time in programmatic settings can be challenging. Despite the positive reception of pooling by laboratory technicians for its time-saving properties [22], pooled testing for TB is not feasible in all laboratories. For example, though specificity issues have not been widely reported, the manipulation of several samples bears the risk of contamination. CXR and AI are also not readily available in most high TB-burden countries. Nevertheless, as pooling becomes established practice in more indications and access to AI improves, this type of pragmatic approach could mitigate commonly encountered cartridge shortages and provide more people with signs of TB access to molecular tests and should be evaluated prospectively to see how they work in real-world settings [39].

## Conclusions

To achieve End TB Strategy goals, it is necessary to optimize the use of available tools. Integrating computer-aided chest radiography and pooling into TB screening and testing algorithms has the potential to substantially reduce diagnostic testing, thus freeing up constrained financial and human public health resources to save costs and extend access to more people in need of high-quality, rapid molecular testing for TB.

### Supplementary Information

Additional file 1: Table S1. Testing data for the four countries across the AI-score deciles. Table S2. Testing threshold information for the four countries across the AI-score deciles. Table S3. Testing combination information for the four countries across the AI-score deciles. Table S4. Results of the sensitivity analysis based on pool size of 3 and different sensitivity and specificity parameters of the pooling method. Table S5. Incremental and cumulative savings by country.

## Data Availability

All the data used for the analysis is available in the paper. The reproducible code and data for the analysis are available in a public GitHub repository at https://github.com/peptonefizz/Xpert-pooling-analysis [39].

## Abbreviations

ACF: Active Case Finding
AI: Artificial Intelligence
CXR: Chest X-ray
mWRD: molecular WHO-recommended Rapid Diagnostic
NAAT: Nucleic Acid Amplification Test
TB: Tuberculosis
WHO: World Health Organization
