# Supplementary material for: Expanding molecular diagnostic coverage for tuberculosis by combining computer-aided chest radiography and sputum specimen pooling: a modeling study from four high-burden countries
Source: BMC Glob Public Health. 2024 Aug 1;2:52. doi: 10.1186/s44263-024-00081-2 (PMC11291606; doi:10.1186/s44263-024-00081-2)
Supplement: Supplementary file 1 — Additional file 1: Table S1. Testing data for the four countries across the AI-score deciles. Table S2. Testing threshold information for the four countries across the AI-score deciles. Table S3. Testing combination information for the four countries across the AI-score deciles. Table S4. Results of the sensitivity analysis based on pool size of 3 and different sensitivity and specificity parameters of the pooling method. Table S5. Incremental and cumulative savings by country. [file 44263_2024_81_MOESM1_ESM.docx]

# Additional file 1

## Table S1: Testing data for the four countries across the AI-score deciles

| **AI-score decile** | **qXR score** | **Bangladesh (qXRv3)** | | **Nigeria**  **(qXRv3)** | | **Viet Nam (qXRv3)** | |  | **CAD4TB score** | **Bangladesh**  **(CAD4TB6)** | | **Zambia**  **(CAD4TB7)** | |
| --- | --- | --- | --- | --- | --- | --- | --- | --- | --- | --- | --- | --- | --- |
|  |  | Tests | Positive | Tests | Positive | Tests | Positive |  |  | Tests | Positive | Tests | Positive |
| *D1* | 0–0.09 | 9774 | 71 | 178 | 1 | 1123 | 5 |  | 0-9 | 10588 | 58 | 143 | 1 |
| *D2* | 0.10–0.19 | 1830 | 46 | 46 | 1 | 465 | 5 |  | 10-19 | 1741 | 110 | 46 | 5 |
| *D3* | 0.20–0.29 | 1027 | 43 | 59 | 2 | 295 | 5 |  | 20-29 | 1024 | 225 | 46 | 3 |
| *D4* | 0.30–0.39 | 982 | 56 | 155 | 2 | 274 | 8 |  | 30-39 | 915 | 212 | 54 | 14 |
| *D5* | 0.40–0.49 | 942 | 53 | 153 | 3 | 265 | 12 |  | 40-49 | 1021 | 750 | 82 | 41 |
| *D6* | 0.50–0.59 | 1006 | 96 | 94 | 9 | 331 | 18 |  | 50-59 | 892 | 411 | 80 | 22 |
| *D7* | 0.60–0.69 | 1104 | 136 | 62 | 3 | 352 | 22 |  | 60-69 | 877 | 199 | 103 | 27 |
| *D8* | 0.70–0.79 | 1676 | 276 | 45 | 5 | 402 | 22 |  | 70-79 | 1011 | 191 | 175 | 51 |
| *D9* | 0.80–0.89 | 3183 | 1114 | 72 | 14 | 635 | 73 |  | 80-89 | 1342 | 111 | 283 | 53 |
| *D10* | 0.90–0.99 | 2555 | 1788 | 157 | 45 | 932 | 285 |  | 90-99 | 4668 | 86 | 2667 | 56 |
|  | **Total** | 24079 | 3679 | 1021 | 85 | 5074 | 455 |  |  | 24079 | 2353 | 3679 | 273 |

### Table S1 note

The column “Tests” and “Positive” indicate total tests performed and positive tests out of the tests performed, respectively. For Bangladesh, both qXRv3 and CAD4TB6 readings were available for the same dataset with the former used in the main analysis.

## Table S2: Testing threshold information for the four countries across the AI-score deciles

| **AI-score decile** | **qXR score** | **Bangladesh**  **(qXRv3)** | **Nigeria**  **(qXRv3)** | **Viet Nam**  **(qXRv3)** |  | **CAD4TB score** | **Bangladesh**  **(CAD4TB6)** | **Zambia**  **(CAD4TB7)** |
| --- | --- | --- | --- | --- | --- | --- | --- | --- |
| *D1* | 0–0.09 | No testing | No testing | No testing |  | 0-9 | No testing | No testing |
| *D2* | 0.10–0.19 | No testing | No testing | No testing |  | 10-19 | No testing | No testing |
| *D3* | 0.20–0.29 | No testing | No testing | No testing |  | 20-29 | Testing | No testing |
| *D4* | 0.30–0.39 | Testing | Testing | No testing |  | 30-39 | Testing | Testing |
| *D5* | 0.40–0.49 | Testing | Testing | Testing |  | 40-49 | Testing | Testing |
| *D6* | 0.50–0.59 | Testing | Testing | Testing |  | 50-59 | Testing | Testing |
| *D7* | 0.60–0.69 | Testing | Testing | Testing |  | 60-69 | Testing | Testing |
| *D8* | 0.70–0.79 | Testing | Testing | Testing |  | 70-79 | Testing | Testing |
| *D9* | 0.80–0.89 | Testing | Testing | Testing |  | 80-89 | Testing | Testing |
| *D10* | 0.90–0.99 | Testing | Testing | Testing |  | 90-99 | Testing | Testing |

### Table S2 note

The columns with information on “Testing” and “No testing” indicate the AI-score deciles where testing was not performed based on the threshold defined in the methods to achieve 5% missed cases overall or closest thereof as a result of not employing Xpert testing in the previous deciles. For Bangladesh, both qXRv3 and CAD4TB6 readings were available for the same dataset with the former used in the main analysis.

## Table S3: Testing combination information for the four countries across the AI-score deciles

| **Country** | **Platform** | **CAD score** | **CXR Case** | **General Pooling Case** | **AI-guided Pooling Case** | **AI-guided CAD Cohort Pooling Case** |
| --- | --- | --- | --- | --- | --- | --- |
| Bangladesh | qXRv3 | 0–0.09 | No | No | No | No |
| Bangladesh | qXRv3 | 0.10–0.19 | No | No | No | No |
| Bangladesh | qXRv3 | 0.20–0.29 | No | No | No | No |
| Bangladesh | qXRv3 | 0.30–0.39 | Individual | Pool 1 | Pool 1 | Pool 1 |
| Bangladesh | qXRv3 | 0.40–0.49 | Individual | Pool 1 | Pool 1 | Pool 1 |
| Bangladesh | qXRv3 | 0.50–0.59 | Individual | Pool 1 | Pool 1 | Pool 1 |
| Bangladesh | qXRv3 | 0.60–0.69 | Individual | Pool 1 | Pool 1 | Pool 2 |
| Bangladesh | qXRv3 | 0.70–0.79 | Individual | Pool 1 | Pool 1 | Pool 2 |
| Bangladesh | qXRv3 | 0.80–0.89 | Individual | Pool 1 | Individual | Individual |
| Bangladesh | qXRv3 | 0.90–0.99 | Individual | Pool 1 | Individual | Individual |
| Bangladesh | CAD4TB6 | 0-9 | No | No | No | No |
| Bangladesh | CAD4TB6 | 10-19 | No | No | No | No |
| Bangladesh | CAD4TB6 | 20-29 | Individual | Pool 1 | Pool 1 | Pool 1 |
| Bangladesh | CAD4TB6 | 30-39 | Individual | Pool 1 | Pool 1 | Pool 1 |
| Bangladesh | CAD4TB6 | 40-49 | Individual | Pool 1 | Pool 1 | Pool 1 |
| Bangladesh | CAD4TB6 | 50-59 | Individual | Pool 1 | Pool 1 | Pool 1 |
| Bangladesh | CAD4TB6 | 60-69 | Individual | Pool 1 | Pool 1 | Pool 2 |
| Bangladesh | CAD4TB6 | 70-79 | Individual | Pool 1 | Pool 1 | Pool 2 |
| Bangladesh | CAD4TB6 | 80-89 | Individual | Pool 1 | Pool 1 | Pool 3 |
| Bangladesh | CAD4TB6 | 90-99 | Individual | Pool 1 | Individual | Individual |
| Nigeria | qXRv3 | 0–0.09 | No | No | No | No |
| Nigeria | qXRv3 | 0.10–0.19 | No | No | No | No |
| Nigeria | qXRv3 | 0.20–0.29 | No | No | No | No |
| Nigeria | qXRv3 | 0.30–0.39 | Individual | Pool 1 | Pool 1 | Pool 1 |
| Nigeria | qXRv3 | 0.40–0.49 | Individual | Pool 1 | Pool 1 | Pool 1 |
| Nigeria | qXRv3 | 0.50–0.59 | Individual | Pool 1 | Pool 1 | Pool 1 |
| Nigeria | qXRv3 | 0.60–0.69 | Individual | Pool 1 | Pool 1 | Pool 1 |
| Nigeria | qXRv3 | 0.70–0.79 | Individual | Pool 1 | Pool 1 | Pool 1 |
| Nigeria | qXRv3 | 0.80–0.89 | Individual | Pool 1 | Pool 1 | Pool 2 |
| Nigeria | qXRv3 | 0.90–0.99 | Individual | Pool 1 | Individual | Pool 2 |
| Viet Nam | qXRv3 | 0–0.09 | No | No | No | No |
| Viet Nam | qXRv3 | 0.10–0.19 | No | No | No | No |
| Viet Nam | qXRv3 | 0.20–0.29 | No | No | No | No |
| Viet Nam | qXRv3 | 0.30–0.39 | No | No | No | No |
| Viet Nam | qXRv3 | 0.40–0.49 | Individual | Pool 1 | Pool 1 | Pool 1 |
| Viet Nam | qXRv3 | 0.50–0.59 | Individual | Pool 1 | Pool 1 | Pool 1 |
| Viet Nam | qXRv3 | 0.60–0.69 | Individual | Pool 1 | Pool 1 | Pool 1 |
| Viet Nam | qXRv3 | 0.70–0.79 | Individual | Pool 1 | Pool 1 | Pool 1 |
| Viet Nam | qXRv3 | 0.80–0.89 | Individual | Pool 1 | Pool 1 | Pool 2 |
| Viet Nam | qXRv3 | 0.90–0.99 | Individual | Pool 1 | Individual | Individual |
| Zambia | CAD4TB7 | 0-9 | No | No | No | No |
| Zambia | CAD4TB7 | 10-19 | No | No | No | No |
| Zambia | CAD4TB7 | 20-29 | No | No | No | No |
| Zambia | CAD4TB7 | 30-39 | Individual | Pool 1 | Pool 1 | Pool 1 |
| Zambia | CAD4TB7 | 40-49 | Individual | Pool 1 | Pool 1 | Pool 1 |
| Zambia | CAD4TB7 | 50-59 | Individual | Pool 1 | Pool 1 | Pool 1 |
| Zambia | CAD4TB7 | 60-69 | Individual | Pool 1 | Pool 1 | Pool 2 |
| Zambia | CAD4TB7 | 70-79 | Individual | Pool 1 | Pool 1 | Pool 2 |
| Zambia | CAD4TB7 | 80-89 | Individual | Pool 1 | Individual | Individual |
| Zambia | CAD4TB7 | 90-99 | Individual | Pool 1 | Individual | Individual |

### Table S3 note

The column with information on testing indicates the testing strategy utilized for each AI-score decile across the four approaches. An additional approach, “AI-guided CAD Cohort Pooling Case”, is also shown that is not included in the main analysis. For Bangladesh, both qXRv3 and CAD4TB6 readings were available for the same dataset with the former used in the main analysis.

## Table S4: Results of the sensitivity analysis based on pool size of 3 and different sensitivity and specificity parameters of the pooling method

#### Bangladesh-qXRv3

| **Country** | **Platform** | **Pool size** | **Pooling case** | **Pooling sensitivity** | **Pooling specificity: 0.98** | **Pooling specificity: 1** | **Comparator (Pooling sensitivity & specificity: 1)** | **Savings in specificity: 0.98** | **Savings in specificity: 1** |
| --- | --- | --- | --- | --- | --- | --- | --- | --- | --- |
| Bangladesh | qXRv3 | Pools of 3 | CXR Case | 0.9 | 11448 | 11448 | 11448 | 0.0% | 0.0% |
| Bangladesh | qXRv3 | Pools of 3 | General Pooling Case | 0.9 | 10773 | 10696 | 11461 | 6.0% | 6.7% |
| Bangladesh | qXRv3 | Pools of 3 | AI-guided Pooling Case | 0.9 | 9215 | 9134 | 9300 | 0.9% | 1.8% |
| Bangladesh | qXRv3 | Pools of 3 | AI-guided CAD Cohort Pooling Case | 0.9 | 9195 | 9114 | 9277 | 0.9% | 1.8% |
| Bangladesh | qXRv3 | Pools of 3 | CXR Case | 0.95 | 11448 | 11448 | 11448 | 0.0% | 0.0% |
| Bangladesh | qXRv3 | Pools of 3 | General Pooling Case | 0.95 | 11154 | 11079 | 11461 | 2.7% | 3.3% |
| Bangladesh | qXRv3 | Pools of 3 | AI-guided Pooling Case | 0.95 | 9298 | 9217 | 9300 | 0.0% | 0.9% |
| Bangladesh | qXRv3 | Pools of 3 | AI-guided CAD Cohort Pooling Case | 0.95 | 9277 | 9195 | 9277 | 0.0% | 0.9% |
| Bangladesh | qXRv3 | Pools of 3 | CXR Case | 1 | 11448 | 11448 | 11448 | 0.0% | 0.0% |
| Bangladesh | qXRv3 | Pools of 3 | General Pooling Case | 1 | 11537 | 11461 | 11461 | -0.7% | 0.0% |
| Bangladesh | qXRv3 | Pools of 3 | AI-guided Pooling Case | 1 | 9381 | 9300 | 9300 | -0.9% | 0.0% |
| Bangladesh | qXRv3 | Pools of 3 | AI-guided CAD Cohort Pooling Case | 1 | 9359 | 9277 | 9277 | -0.9% | 0.0% |
| Bangladesh | qXRv3 | Pools of 4 | CXR Case | 0.9 | 11448 | 11448 | 11448 | 0.0% | 0.0% |
| Bangladesh | qXRv3 | Pools of 4 | General Pooling Case | 0.9 | 10847 | 10795 | 11676 | 7.1% | 7.6% |
| Bangladesh | qXRv3 | Pools of 4 | AI-guided Pooling Case | 0.9 | 9125 | 9053 | 9262 | 1.5% | 2.3% |
| Bangladesh | qXRv3 | Pools of 4 | AI-guided CAD Cohort Pooling Case | 0.9 | 9089 | 9015 | 9221 | 1.4% | 2.2% |
| Bangladesh | qXRv3 | Pools of 4 | CXR Case | 0.95 | 11448 | 11448 | 11448 | 0.0% | 0.0% |
| Bangladesh | qXRv3 | Pools of 4 | General Pooling Case | 0.95 | 11288 | 11236 | 11676 | 3.3% | 3.8% |
| Bangladesh | qXRv3 | Pools of 4 | AI-guided Pooling Case | 0.95 | 9230 | 9157 | 9262 | 0.4% | 1.1% |
| Bangladesh | qXRv3 | Pools of 4 | AI-guided CAD Cohort Pooling Case | 0.95 | 9191 | 9118 | 9221 | 0.3% | 1.1% |
| Bangladesh | qXRv3 | Pools of 4 | CXR Case | 1 | 11448 | 11448 | 11448 | 0.0% | 0.0% |
| Bangladesh | qXRv3 | Pools of 4 | General Pooling Case | 1 | 11729 | 11676 | 11676 | -0.5% | 0.0% |
| Bangladesh | qXRv3 | Pools of 4 | AI-guided Pooling Case | 1 | 9334 | 9262 | 9262 | -0.8% | 0.0% |
| Bangladesh | qXRv3 | Pools of 4 | AI-guided CAD Cohort Pooling Case | 1 | 9294 | 9221 | 9221 | -0.8% | 0.0% |

#### B. Bangladesh-CAD4TB6

| **Country** | **Platform** | **Pool size** | **Pooling case** | **Pooling sensitivity** | **Pooling specificity: 0.98** | **Pooling specificity: 1** | **Comparator (Pooling sensitivity & specificity: 1)** | **Savings in specificity: 0.98** | **Savings in specificity: 1** |
| --- | --- | --- | --- | --- | --- | --- | --- | --- | --- |
| Bangladesh | CAD4TB6 | Pools of 3 | CXR Case | 0.9 | 11750 | 11750 | 11750 | 0.0% | 0.0% |
| Bangladesh | CAD4TB6 | Pools of 3 | General Pooling Case | 0.9 | 10900 | 10819 | 11585 | 5.9% | 6.6% |
| Bangladesh | CAD4TB6 | Pools of 3 | AI-guided Pooling Case | 0.9 | 9101 | 9003 | 9222 | 1.3% | 2.4% |
| Bangladesh | CAD4TB6 | Pools of 3 | AI-guided CAD Cohort Pooling Case | 0.9 | 9049 | 8950 | 9163 | 1.2% | 2.3% |
| Bangladesh | CAD4TB6 | Pools of 3 | CXR Case | 0.95 | 11750 | 11750 | 11750 | 0.0% | 0.0% |
| Bangladesh | CAD4TB6 | Pools of 3 | General Pooling Case | 0.95 | 11284 | 11202 | 11585 | 2.6% | 3.3% |
| Bangladesh | CAD4TB6 | Pools of 3 | AI-guided Pooling Case | 0.95 | 9210 | 9112 | 9222 | 0.1% | 1.2% |
| Bangladesh | CAD4TB6 | Pools of 3 | AI-guided CAD Cohort Pooling Case | 0.95 | 9156 | 9057 | 9163 | 0.1% | 1.2% |
| Bangladesh | CAD4TB6 | Pools of 3 | CXR Case | 1 | 11750 | 11750 | 11750 | 0.0% | 0.0% |
| Bangladesh | CAD4TB6 | Pools of 3 | General Pooling Case | 1 | 11667 | 11585 | 11585 | -0.7% | 0.0% |
| Bangladesh | CAD4TB6 | Pools of 3 | AI-guided Pooling Case | 1 | 9320 | 9222 | 9222 | -1.1% | 0.0% |
| Bangladesh | CAD4TB6 | Pools of 3 | AI-guided CAD Cohort Pooling Case | 1 | 9263 | 9163 | 9163 | -1.1% | 0.0% |
| Bangladesh | CAD4TB6 | Pools of 4 | CXR Case | 0.9 | 11750 | 11750 | 11750 | 0.0% | 0.0% |
| Bangladesh | CAD4TB6 | Pools of 4 | General Pooling Case | 0.9 | 10988 | 10930 | 11819 | 7.0% | 7.5% |
| Bangladesh | CAD4TB6 | Pools of 4 | AI-guided Pooling Case | 0.9 | 9010 | 8924 | 9200 | 2.1% | 3.0% |
| Bangladesh | CAD4TB6 | Pools of 4 | AI-guided CAD Cohort Pooling Case | 0.9 | 8920 | 8831 | 9097 | 2.0% | 2.9% |
| Bangladesh | CAD4TB6 | Pools of 4 | CXR Case | 0.95 | 11750 | 11750 | 11750 | 0.0% | 0.0% |
| Bangladesh | CAD4TB6 | Pools of 4 | General Pooling Case | 0.95 | 11432 | 11374 | 11819 | 3.3% | 3.8% |
| Bangladesh | CAD4TB6 | Pools of 4 | AI-guided Pooling Case | 0.95 | 9149 | 9062 | 9200 | 0.6% | 1.5% |
| Bangladesh | CAD4TB6 | Pools of 4 | AI-guided CAD Cohort Pooling Case | 0.95 | 9053 | 8965 | 9097 | 0.5% | 1.5% |
| Bangladesh | CAD4TB6 | Pools of 4 | CXR Case | 1 | 11750 | 11750 | 11750 | 0.0% | 0.0% |
| Bangladesh | CAD4TB6 | Pools of 4 | General Pooling Case | 1 | 11876 | 11819 | 11819 | -0.5% | 0.0% |
| Bangladesh | CAD4TB6 | Pools of 4 | AI-guided Pooling Case | 1 | 9287 | 9200 | 9200 | -1.0% | 0.0% |
| Bangladesh | CAD4TB6 | Pools of 4 | AI-guided CAD Cohort Pooling Case | 1 | 9185 | 9097 | 9097 | -1.0% | 0.0% |

#### C. Nigeria-qXRv3

| **Country** | **Platform** | **Pool size** | **Pooling case** | **Pooling sensitivity** | **Pooling specificity: 0.98** | **Pooling specificity: 1** | **Comparator (Pooling sensitivity & specificity: 1)** | **Savings in specificity: 0.98** | **Savings in specificity: 1** |
| --- | --- | --- | --- | --- | --- | --- | --- | --- | --- |
| Nigeria | qXRv3 | Pools of 3 | CXR Case | 0.9 | 738 | 738 | 738 | 0.0% | 0.0% |
| Nigeria | qXRv3 | Pools of 3 | General Pooling Case | 0.9 | 453 | 442 | 464 | 2.4% | 4.7% |
| Nigeria | qXRv3 | Pools of 3 | AI-guided Pooling Case | 0.9 | 452 | 442 | 453 | 0.2% | 2.4% |
| Nigeria | qXRv3 | Pools of 3 | AI-guided CAD Cohort Pooling Case | 0.9 | 436 | 426 | 445 | 2.0% | 4.3% |
| Nigeria | qXRv3 | Pools of 3 | CXR Case | 0.95 | 738 | 738 | 738 | 0.0% | 0.0% |
| Nigeria | qXRv3 | Pools of 3 | General Pooling Case | 0.95 | 463 | 453 | 464 | 0.2% | 2.4% |
| Nigeria | qXRv3 | Pools of 3 | AI-guided Pooling Case | 0.95 | 457 | 448 | 453 | -0.9% | 1.1% |
| Nigeria | qXRv3 | Pools of 3 | AI-guided CAD Cohort Pooling Case | 0.95 | 446 | 435 | 445 | -0.2% | 2.3% |
| Nigeria | qXRv3 | Pools of 3 | CXR Case | 1 | 738 | 738 | 738 | 0.0% | 0.0% |
| Nigeria | qXRv3 | Pools of 3 | General Pooling Case | 1 | 474 | 464 | 464 | -2.2% | 0.0% |
| Nigeria | qXRv3 | Pools of 3 | AI-guided Pooling Case | 1 | 462 | 453 | 453 | -2.0% | 0.0% |
| Nigeria | qXRv3 | Pools of 3 | AI-guided CAD Cohort Pooling Case | 1 | 456 | 445 | 445 | -2.5% | 0.0% |
| Nigeria | qXRv3 | Pools of 4 | CXR Case | 0.9 | 738 | 738 | 738 | 0.0% | 0.0% |
| Nigeria | qXRv3 | Pools of 4 | General Pooling Case | 0.9 | 441 | 432 | 459 | 3.9% | 5.9% |
| Nigeria | qXRv3 | Pools of 4 | AI-guided Pooling Case | 0.9 | 430 | 421 | 434 | 0.9% | 3.0% |
| Nigeria | qXRv3 | Pools of 4 | AI-guided CAD Cohort Pooling Case | 0.9 | 414 | 403 | 427 | 3.0% | 5.6% |
| Nigeria | qXRv3 | Pools of 4 | CXR Case | 0.95 | 738 | 738 | 738 | 0.0% | 0.0% |
| Nigeria | qXRv3 | Pools of 4 | General Pooling Case | 0.95 | 455 | 446 | 459 | 0.9% | 2.8% |
| Nigeria | qXRv3 | Pools of 4 | AI-guided Pooling Case | 0.95 | 436 | 427 | 434 | -0.5% | 1.6% |
| Nigeria | qXRv3 | Pools of 4 | AI-guided CAD Cohort Pooling Case | 0.95 | 426 | 415 | 427 | 0.2% | 2.8% |
| Nigeria | qXRv3 | Pools of 4 | CXR Case | 1 | 738 | 738 | 738 | 0.0% | 0.0% |
| Nigeria | qXRv3 | Pools of 4 | General Pooling Case | 1 | 469 | 459 | 459 | -2.2% | 0.0% |
| Nigeria | qXRv3 | Pools of 4 | AI-guided Pooling Case | 1 | 443 | 434 | 434 | -2.1% | 0.0% |
| Nigeria | qXRv3 | Pools of 4 | AI-guided CAD Cohort Pooling Case | 1 | 438 | 427 | 427 | -2.6% | 0.0% |

#### D. Viet Nam-qXRv3

| **Country** | **Platform** | **Pool size** | **Pooling case** | **Pooling sensitivity** | **Pooling specificity: 0.98** | **Pooling specificity: 1** | **Comparator (Pooling sensitivity & specificity: 1)** | **Savings in specificity: 0.98** | **Savings in specificity: 1** |
| --- | --- | --- | --- | --- | --- | --- | --- | --- | --- |
| Viet Nam | qXRv3 | Pools of 3 | CXR Case | 0.9 | 2917 | 2917 | 2917 | 0.0% | 0.0% |
| Viet Nam | qXRv3 | Pools of 3 | General Pooling Case | 0.9 | 2011 | 1975 | 2086 | 3.6% | 5.3% |
| Viet Nam | qXRv3 | Pools of 3 | AI-guided Pooling Case | 0.9 | 1994 | 1962 | 2003 | 0.5% | 2.1% |
| Viet Nam | qXRv3 | Pools of 3 | AI-guided CAD Cohort Pooling Case | 0.9 | 1990 | 1959 | 2000 | 0.5% | 2.1% |
| Viet Nam | qXRv3 | Pools of 3 | CXR Case | 0.95 | 2917 | 2917 | 2917 | 0.0% | 0.0% |
| Viet Nam | qXRv3 | Pools of 3 | General Pooling Case | 0.95 | 2067 | 2031 | 2086 | 0.9% | 2.6% |
| Viet Nam | qXRv3 | Pools of 3 | AI-guided Pooling Case | 0.95 | 2014 | 1983 | 2003 | -0.6% | 1.0% |
| Viet Nam | qXRv3 | Pools of 3 | AI-guided CAD Cohort Pooling Case | 0.95 | 2011 | 1979 | 2000 | -0.6% | 1.1% |
| Viet Nam | qXRv3 | Pools of 3 | CXR Case | 1 | 2917 | 2917 | 2917 | 0.0% | 0.0% |
| Viet Nam | qXRv3 | Pools of 3 | General Pooling Case | 1 | 2122 | 2086 | 2086 | -1.7% | 0.0% |
| Viet Nam | qXRv3 | Pools of 3 | AI-guided Pooling Case | 1 | 2035 | 2003 | 2003 | -1.6% | 0.0% |
| Viet Nam | qXRv3 | Pools of 3 | AI-guided CAD Cohort Pooling Case | 1 | 2031 | 2000 | 2000 | -1.6% | 0.0% |
| Viet Nam | qXRv3 | Pools of 4 | CXR Case | 0.9 | 2917 | 2917 | 2917 | 0.0% | 0.0% |
| Viet Nam | qXRv3 | Pools of 4 | General Pooling Case | 0.9 | 2003 | 1972 | 2110 | 5.1% | 6.5% |
| Viet Nam | qXRv3 | Pools of 4 | AI-guided Pooling Case | 0.9 | 1931 | 1902 | 1955 | 1.2% | 2.7% |
| Viet Nam | qXRv3 | Pools of 4 | AI-guided CAD Cohort Pooling Case | 0.9 | 1925 | 1895 | 1948 | 1.2% | 2.7% |
| Viet Nam | qXRv3 | Pools of 4 | CXR Case | 0.95 | 2917 | 2917 | 2917 | 0.0% | 0.0% |
| Viet Nam | qXRv3 | Pools of 4 | General Pooling Case | 0.95 | 2072 | 2041 | 2110 | 1.8% | 3.3% |
| Viet Nam | qXRv3 | Pools of 4 | AI-guided Pooling Case | 0.95 | 1958 | 1928 | 1955 | -0.2% | 1.4% |
| Viet Nam | qXRv3 | Pools of 4 | AI-guided CAD Cohort Pooling Case | 0.95 | 1950 | 1921 | 1948 | -0.1% | 1.4% |
| Viet Nam | qXRv3 | Pools of 4 | CXR Case | 1 | 2917 | 2917 | 2917 | 0.0% | 0.0% |
| Viet Nam | qXRv3 | Pools of 4 | General Pooling Case | 1 | 2141 | 2110 | 2110 | -1.5% | 0.0% |
| Viet Nam | qXRv3 | Pools of 4 | AI-guided Pooling Case | 1 | 1984 | 1955 | 1955 | -1.5% | 0.0% |
| Viet Nam | qXRv3 | Pools of 4 | AI-guided CAD Cohort Pooling Case | 1 | 1976 | 1948 | 1948 | -1.4% | 0.0% |

#### E. Zambia- CAD4TB7

| **Country** | **Platform** | **Pool size** | **Pooling case** | **Pooling sensitivity** | **Pooling specificity: 0.98** | **Pooling specificity: 1** | **Comparator (Pooling sensitivity & specificity: 1)** | **Savings in specificity: 0.98** | **Savings in specificity: 1** |
| --- | --- | --- | --- | --- | --- | --- | --- | --- | --- |
| Zambia | CAD4TB7 | Pools of 3 | CXR Case | 0.9 | 1960 | 1960 | 1960 | 0.0% | 0.0% |
| Zambia | CAD4TB7 | Pools of 3 | General Pooling Case | 0.9 | 1300 | 1275 | 1344 | 3.3% | 5.1% |
| Zambia | CAD4TB7 | Pools of 3 | AI-guided Pooling Case | 0.9 | 1195 | 1168 | 1211 | 1.3% | 3.6% |
| Zambia | CAD4TB7 | Pools of 3 | AI-guided CAD Cohort Pooling Case | 0.9 | 1181 | 1154 | 1194 | 1.1% | 3.4% |
| Zambia | CAD4TB7 | Pools of 3 | CXR Case | 0.95 | 1960 | 1960 | 1960 | 0.0% | 0.0% |
| Zambia | CAD4TB7 | Pools of 3 | General Pooling Case | 0.95 | 1335 | 1309 | 1344 | 0.7% | 2.6% |
| Zambia | CAD4TB7 | Pools of 3 | AI-guided Pooling Case | 0.95 | 1216 | 1189 | 1211 | -0.4% | 1.8% |
| Zambia | CAD4TB7 | Pools of 3 | AI-guided CAD Cohort Pooling Case | 0.95 | 1201 | 1174 | 1194 | -0.6% | 1.7% |
| Zambia | CAD4TB7 | Pools of 3 | CXR Case | 1 | 1960 | 1960 | 1960 | 0.0% | 0.0% |
| Zambia | CAD4TB7 | Pools of 3 | General Pooling Case | 1 | 1369 | 1344 | 1344 | -1.9% | 0.0% |
| Zambia | CAD4TB7 | Pools of 3 | AI-guided Pooling Case | 1 | 1237 | 1211 | 1211 | -2.2% | 0.0% |
| Zambia | CAD4TB7 | Pools of 3 | AI-guided CAD Cohort Pooling Case | 1 | 1221 | 1194 | 1194 | -2.3% | 0.0% |
| Zambia | CAD4TB7 | Pools of 4 | CXR Case | 0.9 | 1960 | 1960 | 1960 | 0.0% | 0.0% |
| Zambia | CAD4TB7 | Pools of 4 | General Pooling Case | 0.9 | 1287 | 1265 | 1352 | 4.8% | 6.4% |
| Zambia | CAD4TB7 | Pools of 4 | AI-guided Pooling Case | 0.9 | 1151 | 1127 | 1181 | 2.5% | 4.6% |
| Zambia | CAD4TB7 | Pools of 4 | AI-guided CAD Cohort Pooling Case | 0.9 | 1125 | 1100 | 1152 | 2.3% | 4.5% |
| Zambia | CAD4TB7 | Pools of 4 | CXR Case | 0.95 | 1960 | 1960 | 1960 | 0.0% | 0.0% |
| Zambia | CAD4TB7 | Pools of 4 | General Pooling Case | 0.95 | 1331 | 1309 | 1352 | 1.6% | 3.2% |
| Zambia | CAD4TB7 | Pools of 4 | AI-guided Pooling Case | 0.95 | 1178 | 1154 | 1181 | 0.3% | 2.3% |
| Zambia | CAD4TB7 | Pools of 4 | AI-guided CAD Cohort Pooling Case | 0.95 | 1151 | 1127 | 1152 | 0.1% | 2.2% |
| Zambia | CAD4TB7 | Pools of 4 | CXR Case | 1 | 1960 | 1960 | 1960 | 0.0% | 0.0% |
| Zambia | CAD4TB7 | Pools of 4 | General Pooling Case | 1 | 1374 | 1352 | 1352 | -1.6% | 0.0% |
| Zambia | CAD4TB7 | Pools of 4 | AI-guided Pooling Case | 1 | 1206 | 1181 | 1181 | -2.1% | 0.0% |
| Zambia | CAD4TB7 | Pools of 4 | AI-guided CAD Cohort Pooling Case | 1 | 1177 | 1152 | 1152 | -2.2% | 0.0% |

### Table S4 note

The sensitivity analysis is presented in five tables by country and platform combinations. The analysis presents all possible combination of pool sizes (3 or 4), pooling sensitivity of 0.95 or 1, and pooling specificity of 0.98 and 1, across the five approaches (Baseline case, CXR Case, General Pooling Case, AI-guided Pooling Case, AI-guided CAD Cohort Pooling Case). The percentages indicate savings against the baseline case with pooling sensitivity and specificity of 1.

## Table S5: Incremental and cumulative savings by country

|  | **Total tests** | **Incremental savings** | | **Cumulative savings** | |
| --- | --- | --- | --- | --- | --- |
|  | **N** | **N** | **%** | **N** | **%** |
| **Bangladesh-qXRv3** |  |  |  |  |  |
| Baseline case | 24,079 | — | — | — | — |
| CXR approach | 11,448 | 12,631 | 52.5% | 12,631 | 52.5% |
| Indiscriminate pooling approach | 11,676 | -228 | -2.0% | 12,403 | 51.5% |
| AI-guided pooling approach | 9,262 | 2,414 | 20.7% | 14,817 | 61.5% |
| AI-guided CAD cohort pooling case | 9,221 | 41 | **0.4%** | 14,858 | 61.7% |
| **Bangladesh-CAD4TB6** |  |  |  |  |  |
| Baseline case | 24,079 | — | — | — | — |
| CXR approach | 11,750 | 12,329 | 51.2% | 12,329 | 51.2% |
| Indiscriminate pooling approach | 11,819 | -69 | -0.6% | 12,260 | 50.9% |
| AI-guided pooling approach | 9,200 | 2,619 | 22.2% | 14,879 | 61.8% |
| AI-guided CAD cohort pooling case | 9,097 | 103 | **1.1%** | 14,982 | 62.2% |
| **Nigeria-qXRv3** |  |  |  |  |  |
| Baseline case | 1,021 | — | — | — | — |
| CXR approach | 738 | 283 | 27.7% | 283 | 27.7% |
| Indiscriminate pooling approach | 459 | 279 | 37.8% | 562 | 55.0% |
| AI-guided pooling approach | 434 | 25 | 5.4% | 587 | 57.5% |
| AI-guided CAD cohort pooling case | 427 | 7 | **1.6%** | 594 | 58.2% |
| **Viet Nam-qXRv3** |  |  |  |  |  |
| Baseline case | 5,074 | — | — | — | — |
| CXR approach (sensitivity=95.3%) | 2,917 | 2,157 | 42.5% | 2,157 | 42.5% |
| Indiscriminate pooling approach | 2,110 | 807 | 27.7% | 2,964 | 58.4% |
| AI-guided pooling approach | 1,955 | 155 | 7.3% | 3,119 | 61.5% |
| AI-guided CAD cohort pooling case | 1,948 | 7 | **0.4%** | 3,126 | 61.6% |
| **Zambia-CAD4TB7** |  |  |  |  |  |
| Baseline case | 2,353 | — | — | — | — |
| CXR approach | 1,960 | 393 | 16.7% | 393 | 16.7% |
| Indiscriminate pooling approach | 1,352 | 608 | 31.0% | 1,001 | 42.5% |
| AI-guided pooling approach | 1,181 | 171 | 12.6% | 1,172 | 49.8% |
| AI-guided CAD cohort pooling case | 1,152 | 29 | **2.5%** | 1,201 | 51.0% |

### Table S5 note

The model assumes pool sizes of 4 with both pooling and individual testing sensitivity and specificity of 100% for a testing threshold resulting in missed cases of 4.4%, 5.1%, 4.7%, 5.1% and 3.3% for Bangladesh-qXRv3, Bangladesh-CAD4TB6, Nigeria-qXRv3, Viet Nam-qXRv3 and Zambia-CAD4TB7, respectively. Incremental savings indicate the difference from the prior case, whereas cumulative savings are calculated against the baseline approach. This presents results for Bangladesh using both qXRv3 and CAD4TB6, and an additional approach highlighted in red, “AI-guided CAD cohort pooling case” for all countries.
